# Co-infecting Reptarenaviruses Can Be Vertically Transmitted in *Boa Constrictor*

**DOI:** 10.1371/journal.ppat.1006179

**Published:** 2017-01-23

**Authors:** Saskia Keller, Udo Hetzel, Tarja Sironen, Yegor Korzyukov, Olli Vapalahti, Anja Kipar, Jussi Hepojoki

**Affiliations:** 1 Institute of Veterinary Pathology, Vetsuisse Faculty, University of Zürich, Zürich, Switzerland; 2 University of Helsinki, Department of Veterinary Biosciences, Faculty of Veterinary Medicine, Helsinki, Finland; 3 University of Helsinki, Medicum, Department of Virology, Helsinki, Finland; 4 University of Helsinki and Helsinki University Hospital, Department of Virology, Helsinki, Finland; Division of Clinical Research, UNITED STATES

## Abstract

Boid inclusion body disease (BIBD) is an often fatal disease affecting mainly constrictor snakes. BIBD has been associated with infection, and more recently with coinfection, by various reptarenavirus species (family *Arenaviridae*). Thus far BIBD has only been reported in captive snakes, and neither the incubation period nor the route of transmission are known. Herein we provide strong evidence that co-infecting reptarenavirus species can be vertically transmitted in *Boa constrictor*. In total we examined five *B*. *constrictor* clutches with offspring ranging in age from embryos over perinatal abortions to juveniles. The mother and/or father of each clutch were initially diagnosed with BIBD and/or reptarenavirus infection by detection of the pathognomonic inclusion bodies (IB) and/or reptarenaviral RNA. By applying next-generation sequencing and *de novo* sequence assembly we determined the “reptarenavirome” of each clutch, yielding several nearly complete L and S segments of multiple reptarenaviruses. We further confirmed vertical transmission of the co-infecting reptarenaviruses by species-specific RT-PCR from samples of parental animals and offspring. Curiously, not all offspring obtained the full parental “reptarenavirome”. We extended our findings by an *in vitro* approach; cell cultures derived from embryonal samples rapidly developed IB and promoted replication of some or all parental viruses. In the tissues of embryos and perinatal abortions, viral antigen was sometimes detected, but IB were consistently seen only in the juvenile snakes from the age of 2 mo onwards. In addition to demonstrating vertical transmission of multiple species, our results also indicate that reptarenavirus infection induces BIBD over time in the offspring.

## Author Summary

Members of the genus *Reptarenavirus* are “newcomers” of the family *Arenaviridae* and have been associated with boid inclusion body disease (BIBD), an economically important, fatal disease of captive boid snakes. Recently, we and others observed that snakes with BIBD commonly harbour several S and L segments (arenaviruses have a bisegmented genome), which we refer to as co-infection. The above renders reptarenaviruses rather unique and a model for studying viral co-infection. We herein report that reptarenaviruses, and remarkably a whole set of co-infecting reptarenavirus species (based on the nucleotide difference in the L segment), can be transmitted vertically i.e. from parents to offspring. While the parental animals had BIBD, we did not find evidence of the intracytoplasmic inclusions characteristic to BIBD in the infected embryos and perinatal abortions. However, we could confirm the development of BIBD in offspring from an age of 2 months. Our findings further suggest that vertical transmission can, and likely has, significantly influence(d) the evolution of reptarenaviruses, since co-infection will allow reassortment of the viral genomes.

## Introduction

Boid inclusion body disease (BIBD) is a transmissible, progressive and generally fatal disease of boid snakes. First described in the 1970s, BIBD subsequently emerged as a major problem in boid snake collections worldwide [1, 2]. Several genera of boid species have been reported as susceptible to the disease, but its prevalence among snakes as well as its potential occurrence in wild populations is yet unknown [3]. Clinically, BIBD is highly variable particularly in boas, where affected animals can be free of clinical signs, die from secondary infections, or develop neurological signs. The latter are generally more pronounced in pythons. The hallmark of BIBD are the characteristic intracytoplasmic electron dense inclusion bodies (IB) that are found in most cell types [1, 2, 4, 5]. The pathogenesis of BIBD is not yet characterized, and both subclinical as well as chronic disease has been described [2, 6].

A few years ago a novel group of arenaviruses were identified in and isolated from snakes with BIBD [4, 5, 7]. Arenaviruses are negative-sense RNA viruses with two genome segments, L and S, which encode Z protein and RNA-dependent RNA polymerase, and glycoprotein precursor and nucleoprotein (NP), respectively [8]. Strong evidence of the causative relationship between reptarenavirus infection and BIBD is provided by the ability of reptarenavirus isolates to induce the pathognomonic IB in an *in vitro* model [4], and by the fact that the IB contain or mainly consist of reptarenavirus NP [4, 5, 9]. The identification of BIBD-associated arenaviruses led to the formation of a new genus, *Reptarenavirus*, in the family *Arenaviridae*, placing the previously known arenaviruses to another new genus, *Mammarenavirus* [8]. More recently, we and others observed that snakes with BIBD often carry numerous distinct L and S segments, up to four S and 11 L segments were found in a single snake [10, 11]. The taxonomic classification of reptarenaviruses is currently under debate, and in this report we refer to the different L segments as representatives of different reptarenavirus species (species share <76% identity in the L segment [8]). The genomes of reptarenaviruses are highly variable [4, 5, 7, 10–13], as a consequence, the diagnosis of BIBD still relies mainly on the detection of IB in cells in tissues or in blood smears by light microscopy. A recent study screened a large panel of blood samples from captive boid snakes, and found 19% of the snakes to be infected with reptarenavirus [14]. Among *Boa constrictors*, 41.5% were infected, and 87% of the infected snakes were clinically healthy [14]. The authors also compared various detection techniques and found immunohistochemistry (IHC) on blood cells, using a monoclonal anti-reptarenavirus NP antibody, to provide results comparable to hematoxylin-eosin (HE) stained peripheral white blood cells as the standard of diagnosis [14]. However, a few BIBD positive (3/25) samples were negative in IHC [14], which provides further evidence of the high variability among reptarenaviruses.

So far, the route of transmission and the incubation period of reptarenaviruses are unknown, and direct contact or vector mediated transmission by snake mites (*Ophionyssus natricis*) have been proposed [1, 15]. In line with the “transmission through a vector” hypothesis, we recently reported the growth of reptarenaviruses also in arthropod cell lines [15]. Vertical transmission is defined as any transfer of an infectious agent from one generation to the next, including transmission through gametes (i.e. oocyte or spermatocyte), transplacental transmission or perinatal infections [16]. Mammarenaviruses can be vertically transmitted in their reservoir rodent hosts [17–20]. Prenatal infection plays an important role in arenavirus maintenance, and, at least in the case of Lymphocytic choriomeningitis virus (LCMV), Machupo virus (MACV) and Lassa virus (LASV), leads to chronic infection [21].The vertical transmission of BIBD from dam to offspring in both egg-lying and live-bearing snakes has been considered by Chang and Jacobson [1]. Reptiles are divided into oviparous (egg layer) or viviparous (live bearers) species. They represent an important phylogenetic intermedium between anamniotes and amniote vertebrates, displaying all three embryonic membranes: the chorion, allantois and amnion [22]. Viviparous snakes, including *B*. *contrictor*, have a simple placenta that is responsible for gas exchange, and water and nutrient supply [23]. A thin eggshell exists between foetal and maternal placenta but it deteriorates in late gestation, allowing direct contact between the foetal (chorioallantois) and maternal (uterine epithelium) placenta [23]. Normally foetal and maternal epithelia remain intact and the maternal and fetal blood does not mix. Studies on the vertical transmission in reptiles are scarce and include only few viruses, such as equine encephalitis virus [24], adenovirus [25]; Herpesvirus M [26, 27] and, very recently, Sunshinevirus [28].

We set up this study to determine whether reptarenaviruses can be vertically transmitted. For this purpose, five *B*. *constrictor* clutches, represented by parental animals diagnosed with BIBD by traditional methods, or RT-PCR positive for reptarenavirus, and their offspring, ranging from embryos in the first trimester to 20-month-old juveniles, were examined. We applied next-generation sequencing (NGS) to identify the reptarenaviruses of each clutch, which we will refer to as the “reptarenavirome” throughout the manuscript. We utilized virus-specific RT-PCRs to confirm the reptarenavirus L and S segments identified by NGS. Primary cell cultures originating from the embryos served to evaluate the potential of the infecting viruses to induce IB formation and thereby also the disease.

## Results

### BIBD and reptarenavirus infection in the parents and offspring

**Fig 1:**
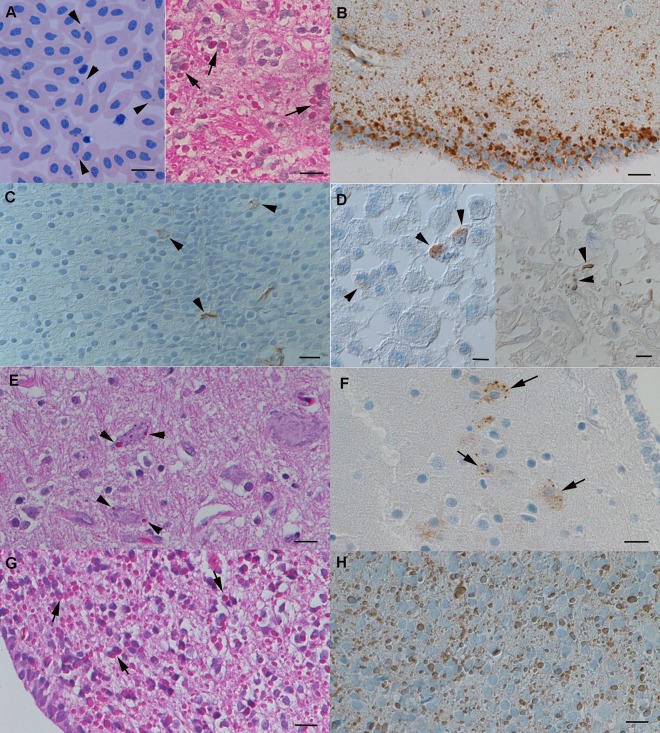
Confirmation of BIBD in parental animals and offspring. **A, B**. Clutch 1, BIBD-positive mother. **A**. The characteristic cytoplasmic inclusion bodies (IB; arrows) are present in erythrocytes (left, blood smear, May-Grünwald Giemsa stain) and in cells in tissues (brain). **B**. Immunohistology confirms the presence of abundant reptarenavirus antigen in all cell types in association with the presence of the IB. **C.** Clutch 2, embryo (E2.1). A few neurons in the spinal cord exhibit reptarenaviral antigen in the cytoplasm (arrowheads). **D**. Clutch 2, embryo (E2.1). Cell pellet from a brain cell culture. Left: passage 1, right: passage 6. There are individual cells expressing viral antigen (arrowheads). **E, F.** Clutch 3, perinatal abortion (PNA2,2), brain. **E**. A few individual neurons exhibit BIBD IBs (arrows). **F**. Reptarenavirus antigen expression is seen in association with inclusion bodies and dispersed in the cytoplasm (arrows). **G, H**. Clutch 5, juvenile (J5.4), 8 mo, brain. **G**. Abundant IB (arrows) are seen within almost all cells. **H**. Reptarenavirus antigen expression is seen in association with inclusion bodies. A, E, G: haematoxylin eosin stain; B, D, F, H: HRP method, haematoxylin counterstain. Bars = 10 μm.

The diagnosis of BIBD is confirmed when the characteristic eosinophilic cytoplasmic IB are seen within cells. These IB contain abundant reptarenavirus NP which can be visualised by immunohistology (IH) [4, 5, 9]; RT-PCR can serve to confirm reptarenavirus infection. We verified the parental animals as BIBD positive and/or positive for reptarenavirus infection using histology and IH. The detection of the IB in cells in cytological and/or histological specimens is currently the widely accepted gold standard for the diagnosis of BIBD, since the IB are pathognomonic; IH confirms the presence of reptarenavirus NP in the cells [4]. For clutches 1 and 3–5 histology and IH were complemented by RT-PCR, which was set up at the time when only four reptarenaviruses were fully sequenced (referred to as “initial RT-PCR”); it targets the L segment of GGV, UHV, and Boa AV NL B3 (Table 1 and Fig 1A and 1B). Interestingly, the blood of both parental animals in clutch 4 was RT-PCR-positive, but no IB were detected in blood cells. However, the subsequent post mortem analysis of the father revealed IB formation and expression of viral antigen in tissues, confirming BIBD (Table 1).

**Table 1:** Clutches, animals and tests performed on individual animals. Summary of results obtained for each animal.

Animals (age)	Cytoplasmic IB/viral antigen			Nucleic acid analysis	
	Blood Cytology	PM Histology	Cell Culture	Initial RT-PCR [specimen]	NGS specimen(s)
**Clutch 1, Breeder 1**					
Mother	**Pos**	**Pos**^IB,^ ^A^ ^[^^1^^–^^7^^]^		**Pos** [brain]	Brain
E1.1		**Pos**^**A**^ ^[^^1^^,^ ^3^^,^ ^4^^,^ ^6^^]^		**Pos** [head]	Head
E1.2		**Pos**^**A**^ ^[^^1^^,^ ^3^^,^ ^4^^,^ ^6^^]^		Neg [body]	Body
E1.3		**Pos**^**A**^ ^[^^1^^,^ ^3^^,^ ^4^^,^ ^6^^]^			
E1.4		**Pos**^**A**^ ^[^^1^^,^ ^3^^,^ ^4^^,^ ^6^^]^			
E1.5		**Pos**^**A**^ ^[^^1^^,^ ^3^^,^ ^4^^,^ ^6^^]^			
E1.6			Neg^B^	**Pos** [CCS]	CCS
E1.7			**Pos**^B^	**Pos** [CCS]	CCS
**Clutch 2, Breeder 2**					
Mother	Neg	**Pos**^IB,^ ^A^ ^[^^1^^–^^7^^]^			Brain
Father^C^	Neg				
E2.1			**Pos**^B^	**Pos** [CCS]	Placenta, CC
E2.2			**Pos**^B^	**Pos** [CCS]	Body, CC
E2.3		**Pos**^**A**^ ^[^^1^^,^ ^3^^,^ ^4^^]^			Body
**Clutch 3, Breeder 1**					
Mother^C^	**Pos**			**Pos** [blood]	Serum
PNA3.1		Neg^IB,^^A^		**Pos** [brain]	Brain
PNA3.2		**Pos**^IB,^ ^A^ ^[^^1^^,^ ^4^^]^		**Pos** [brain]	Brain
PNA3.3		Neg^IB,^^A^		Neg [brain]	Brain
J3.1 (2 mo)		**Pos**^IB,^ ^A^ ^[^^1^^,^ ^2^^,^ ^4^^–^^7^^]^		**Pos** [brain]	
J3.2 (2 mo)		Neg^IB,^^A^		**Pos** [brain]	
**Clutch 4, Breeder 1**					
Mother^C^	Neg			**Pos** [blood]	Serum
Father	Neg	**Pos**^IB,^ ^A^ ^[^^1^^–^^8^^]^		**Pos** [blood]	Serum, lung
PNA4.1		Neg^IB,^^A^		**Pos** [brain]	Placenta, organs
PNA4.2		Neg^IB,^^A^		**Pos** [lung]	
**Clutch 5, Breeder 1**					
Father		**Pos**^IB,^ ^A^ ^[^^1^^,^ ^2^^,^ ^4^^–^^7^^]^		**Pos** [brain]	
J5.1 (8 mo)	**Pos**	**Pos**^IB,^ ^A^ ^[^^1^^–^^7^^]^		Neg [blood]	
J5.2 (8 mo)		**Pos**^IB,^ ^A^ ^[^^1^^–^^8^^]^		Neg [blood]	
J5.3 (8 mo)		**Pos**^IB,^ ^A^ ^[^^1^^–^^8^^]^		Neg [blood]	
J5.4 (8 mo)		**Pos**^IB,^ ^A^ ^[^^1^^–^^8^^]^		**Pos** [blood]	
J5.5 (8 mo)		Neg^IB,^^A^		Neg [blood]	
J5.6 (8 mo)		**Pos**^IB,^ ^A^ ^[^^1^^–^^6^^,^ ^8^^]^		**Pos** [blood]	
J5.7 (8 mo)		**Pos**^IB,^ ^A^ ^[^^1^^,^ ^2^^,^ ^4^^–^^8^^]^		**Pos** [blood]	
J5.8 (12 mo)	Neg	Neg^IB^/(**pos**)^**A**^		**Pos** [brain]	
J5.9 (12 mo)	**Pos**	**Pos**^IB,^ ^A^ ^[^^1^^–^^8^^]^		**Pos** [brain]	
J5.10 (12 mo)	**Pos**	**Pos**^IB,^ ^A^ ^[^^1^^–^^8^^]^		**Pos** [brain]	
J5.11 (12 mo)	Neg	Neg^IB^/(**pos**)^**A**^		**Pos** [brain]	
J5.12 (12 mo)	**Pos**	**Pos**^IB,^ ^A^ ^[^^1^^–^^8^^]^		**Pos** [brain]	
J5.13 (12 mo)	**Pos**	**Pos**^IB,^ ^A^ ^[^^1^^–^^8^^]^		**Pos** [brain]	
J5.14 (12 mo)	**Pos**	**Pos**^IB,^ ^A^ ^[^^1^^–^^3^^,^ ^5^^–^^8^^]^		**Pos** [brain]	
J5.15 (12 mo)	**Pos**	**Pos**^IB,^ ^A^ ^[^^1^^–^^8^^]^		**Pos** [brain]	
J5.16 (12 mo)	**Pos**	**Pos**^IB,^ ^A^ ^[^^1^^,^ ^3^^–^^8^^]^		**Pos** [brain]	
J5.17 (12 mo)	**Pos**	**Pos**^IB,^ ^A^ ^[^^1^^,^ ^3^^–^^8^^]^		**Pos** [brain]	
J5.18 (12 mo)	**Pos**	**Pos**^IB,^ ^A^ ^[^^1^^–^^8^^]^		**Pos** [brain]	
J5.19 (18 mo)		**Pos**^IB,^ ^A^ ^[^^1^^–^^8^^]^		**Pos** [brain]	
J5.20 (18 mo)		**Pos**^IB,^ ^A^ ^[^^1^^,^ ^3^^–^^8^^]^		**Pos** [brain]	
J5.21 (20 mo)	**Pos**	**Pos**^IB,^ ^A^ ^[^^1^^–^^8^^]^		**Pos** [blood]	
J5.22 (20 mo)	**Pos**	**Pos**^IB,^ ^A^ ^[^^1^^–^^8^^]^		**Pos** [blood]	

IB—inclusion bodies (as seen in HE stained tissue section or in May Grünwald-Giemsa stained blood smear), PM—post mortem, E—embryo, PNA—perinatal abortion, J—juvenile; CC—cell culture for virus isolation; CCS—supernatant from CC; RT-PCR—reverse transcriptase polymerase chain reaction, NGS—next-generation sequencing.

Pos–positive; (pos)–questionable positive; Neg–negative; blank box–not available/not examined

A–tested in tissues by immunohistology

B—tested on formalin-fixed, paraffin-embedded tissue culture pellets by immunocytology

C—Animal still alive.

Tissues tested positive by immunohistology: 1 –brain, 2 –heart, 3 –lung, 4 –liver, 5 –pancreas, 6 –kidney, 7 –spleen, 8 –spinal cord.

For clutch 1, comprised of seven embryos in late first trimester (age determined based on the body length of 15 to 17 cm), five embryos were processed for (immuno)histological examination. These did not exhibit IB formation, but exhibited weak reptarenavirus antigen expression in occasional cells in brain, liver and kidneys (Fig 1C). The remaining two embryos (E1.6 and E1.7, Table 1) were used to establish primary cell cultures. These showed viral antigen expression, but no distinct IB formation (Fig 1D; Table 1). The initial RT-PCR showed the presence of reptarenavirus RNA in the mother and embryos E1.1, E1.6, and E1.7. (Table 1).

For clutch 2 (early first trimester embryos with a body length of 5–6 cm), similar results were obtained. Two of the three embryos (E2.1 and E2.2) were used to establish primary cell cultures, which also showed viral antigen expression but no IB. The cell cultures remained persistently infected throughout the study period as confirmed by the expression of viral RNA and antigen. The third embryo (E2.3) was processed for histology and did not exhibit IB but showed occasional weak viral antigen expression in the brain (Table 1).

Clutch 3 comprised five animals, three of which had been perinatally aborted (PNA3.1 to 3.3). Two of these (PNA3.1 and 3.2) were tested reptarenavirus RNA positive, using the initial RT-PCR on the brain, and one (PNA3.2) exhibited IB and reptarenavirus antigen in the tissues (Fig 1E and 1F). The remaining two animals (J3.1 and 3.2) were euthanized as juveniles two months later. Both were tested positive by the initial RT-PCR on the brain and one also exhibited IB and reptarenavirus antigen in tissues (Table 1).

The two perinatal abortions of clutch 4 were shown to be infected, using the initial RT-PCR, but did not exhibit IB formation or reptarenavirus antigen expression.

Clutch 5 comprised 21 animals. Of the seven juveniles euthanized at the age of eight months, six were diagnosed with BIBD, based on the detection of IB and viral antigen in all examined tissues (Fig 1G and 1H), and three of these (3/6) were found positive in the blood by the initial RT-PCR. At the time of euthanasia the samples were collected purely for diagnostic purposes, and unfortunately no samples were stored for RNA isolation. The remaining (1/7) animal (J5.5) was negative in all these tests. Another 11 siblings were euthanized at the age of 12 months. In nine of these, BIBD was confirmed, with the presence of IB and reptarenavirus antigen in tissue and blood cells and a positive result in the initial RT-PCR. Two (2/11) (J5.8, J5.11) were BIBD-negative, but RT-PCR positive in the brain (Table 1). The last four (4/21) animals were kept by the breeder until they were euthanized at the age of 18 mo (n = 2) and 20 mo (n = 2) due to the breeder’s concern that they suffered from BIBD. These all tested positive for BIBD by histology, IH and initial RT-PCR (Table 1).

### Confirmation of the vertical transmission by next-generation sequencing (NGS) of total RNA isolated from the parents and/or offspring

**Fig 2:**
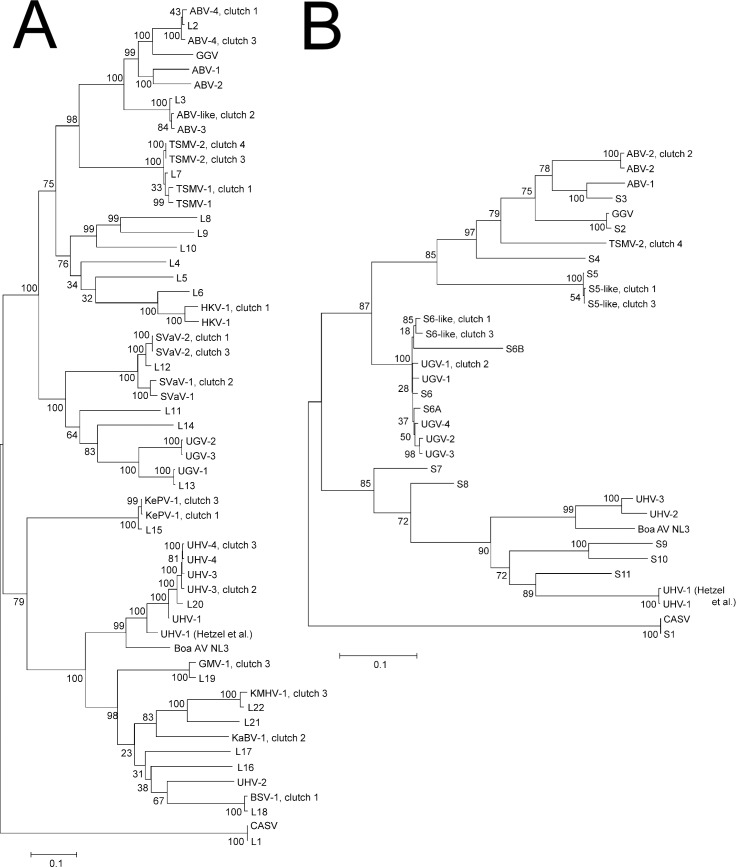
Evolutionary relationships of the reptarenaviruses sequenced in this study. **A**) A Maximum-likelihood tree built on RdRp nt sequences or **B**) complete S segment nt sequences. For simplicity only a single representative of each L or S segment described in [10] is shown. The abbreviations and accession codes are listed in materials and methods. Bootstrap support values are shown at the nodes.

The primers used for RT-PCR in the preliminary screening were designed for the detection of a subset of reptarenaviruses (GGV, UHV, and Boa AV NL B3) at a time when only four reptarenaviruses were known. Subsequently, we and others [10, 11] observed that snakes with BIBD are often co-infected with multiple reptarenavirus species. Therefore, we decided to utilise NGS for further analyses. The NGS study included the first four clutches, but was limited to the animals of which frozen material was available (Table 1). We removed the reads matching a known snake genome (*Python bivitattus*) from the NGS data and performed *de novo* genome assembly. The generated contigs were checked using BLAST (Basic Local Alignment Search Tool, https://blast.ncbi.nlm.nih.gov/Blast.cgi), and although occasional hits to bacterial sequences were identified in some samples, only reptarenavirus sequences were consistently recovered. Similarly to our earlier observation [11], several full-length or almost full-length (at maximum some 200–300 nt missing) reptarenavirus L and S segments were recovered from the parental samples. The coverages (Bowtie 2, [29]) of the reptarenavirus L and S segments derived from the parental animals were >10 (lower coverage at the very last ~50 nts) (S2 Table). In parental animals from breeder 1 (clutches 1 and 3), the following results were obtained: The mother of clutch 1 was positive for six L (Aurora borealis virus-4, ABV-4, GenBank accession KX527594; Tavallinen suomalainen mies virus-1, TSMV-1, KX527595; Hans Kompis virus-1, HKV-1, KX527596; Keijut pohjoismaissa virus-1, KePV-1, KX527597; Bis spöter virus-1, BSV-1, KX527598; Suri Vanera virus, SVaV-2, KX527599) and two S (S6-like, KX527580; S5-like, KX527581) segments, and the mother of clutch 3 was positive for seven L (SVaV-2, KX527587; Kuka mitä häh virus-1, KMHV-1, KX527588; KePV-1, KX527589; University of Helsinki virus-4, UHV-4, KX527590; TSMV-2, KX527591; ABV-4, KX527592; Grüetzi mitenand virus-1, GMV-1, KX527593) and two S (S6-like, KX527578; S5-like, KX527579) segments. Curiously, the brain of the father of clutch 4 was positive for only one pair of L (TSMV-2, KX527582) and S (TSMV-2, KX527575) segments, whereas no reptarenavirus genomes were recovered by NGS from the mother despite clear evidence of BIBD (Table 1). The mother of clutch 2 owned by breeder 2 was positive for four L (ABV-3, KX527583; Kaltenbach virus-1, KaBV-1, KX527584; SVaV-1, KX527585; UHV-3, KX527586) and two S (ABV-2, KX527576; University of Giessen virus-1-like, UGV-1-like, KX527577) segments, whereas no reptarenavirus genomes were recovered from the serum of the father, whose blood cells were also found negative for IB in the cytological examination, providing further evidence that he was indeed not infected at all. The NGS results for the different clutches are summarized in Table 2 and a phylogenetic tree of the *de novo* assembled L and S segments with database sequences is shown in Fig 2A and 2B. The phylogeny indicates that the reptarenaviromes of the two snake collections (which never exchanged animals; personal communication) share some common species but also comprise unique viruses.

**Table 2:** Detailed results obtained from the different specimens used for the RT-PCR approach to identify L segments (abbreviations and accession codes are given in S1 Table, and in materials and methods) in each animal in clutches 1–5. The virus genomes *de novo* assembled from NGS data of the parental sample and confirmed by VSS RT-PCR indicated by virus abbreviations in bold, and viruses detected by VSS RT-PCR are shown in italics in parents and with + sign in the offspring. +/- sign indicates an ambiguous RT-PCR result.

Animals	Specimen	Viruses detected by virus species-specific (VSS) RT-PCR									
		ABV	SVaV	TSMV	UHV-1/4	KePV-1	BSV-1	GMV-1	KMHV-1	HKV	KaBV-1
**Clutch 1, Breeder 1**											
Mother	Brain	**ABV**	**SVaV**	**TSMV**		**KePV-1**	**BSV-1**			**HKV**	
E1.1	Head	+	+	+		+	+			+	
E1.2	Body	-	-	+/-		+	+			-	
E1.6	CC (brain)	-	-	+/-		+	-			-	
E1.7	CC (brain)	-	-	+		+	+/-			-	
**Clutch 2, Breeder 2**											
Mother	Brain	**ABV-3**	**SVaV**		**UHV-1/4**						**KaBV-1**
Father	Blood	+/-	-		-						-
E2.1	Salpinx	+	+		+						+
	CC (umbilicus)	+	+		-						+
	CC (kidney)	-	-		-						-
	CC (heart)	+	-		-						+/-
E2.2	Body	+/-	+		+						+
	CC (umbilicus)	+	+		+						+
	CC (placenta)	+	+		-						+
	CC (kidney)	+	+		-						+
	CC (liver)	+	+		-						-
E2.3	Body	+/-	+		+						+/-
**Clutch 3, Breeder 1**											
Mother	Blood	**ABV**	**SVaV**	**TSMV**	**UHV-1/4**	**KePV-1**		**GMV-1**	**KMHV-1**		
PNA3.1	Brain	-	-	-	-	-		-	-		
	Kidney	+/-	-	-	-	+		-	-		
	Liver	+	-	-	-	-		+/-	+/-		
PNA3.2	Brain	+	-	-	-	+		-	-		
	Kidney	+	-	-	-	-		+/-	+/-		
	Liver	+	-	-	-	+		+/-	+/-		
PNA3.3	Brain	+/-	-	-	-	+		-	-		
	Kidney	+	-	-	-	+		+/-	-		
	Liver	-	-	-	-	-		+/-	-		
J3.1 (2 mo)	Liver	-	-	+	-	-		+	-		
	Kidney	-	-	+	-	-		+/-	-		
J3.2 (2 mo)	Liver	-	-	-	-	-		-	-		
	Kidney	-	+	+	-	+		+	-		
**Clutch 4, Breeder 1**											
Father	Blood	*ABV-3*	*SVaV*	*TSMV-2*	*UHV-1/4*		*BSV-1*	*GMV-1*	*KMHV-1*		
	Brain	-	-	**TSMV-2**	-		-	-	-		
	Lung	+/-	**+**	-	**+**		+	+	+/-		
Mother	Serum	+/-	**+**	-	**-**		+	-	-		
PNA4.1	Placenta	+/-	**+**	+/-	**-**		+	-	+/-		
	Kidney	+	+/-	+/-	-		+	+	+/-		
	Lung	+	+/-	+/-	+/-		+	-	+/-		
PNA4.2	Placenta	+	-	+/-	^-^		+	+	+/-		
	Kidney	+	-	+/-	^-^		+	-	+/-		
	Lung	+	-	+/-	^-^		+	-	+/-		
	Brain	+/-	+/-	+/-	**-**		-	-	+/-		
**Clutch 5, Breeder 1**											
Father	Blood	*ABV*	*SVaV*	*TSMV-2*	*UHV-1/4*	*KePV-1*					
	Liver	+	**+**	+/-	+/-	+					
J5.8 (12 mo)	Brain	+	-	-	-	+					
J5.8 (12 mo)	Liver	+/-	-	-	-	-					
J5.9 (12 mo)	Liver	+	-	+/-	+	+					
J5.10 (12 mo)	Liver	+	+	+	+	+					
J5.11 (12 mo)	Liver	+	+	-	+	+					
J5.12 (12 mo)	Liver	+	+	+	+	+					
J5.13 (12 mo)	Liver	+	+	+	-	+					
J5.14 (12 mo)	Liver	+	-	-	-	+					
J5.15 (12 mo)	Brain	+	+	+	+	+					
J5.18 (12 mo)	Liver	**+**	-	-	-	+					

Bold–L segments derived by NGS and de novo assembly; blank box–not tested.

Initially *de novo* assembly was attempted for several embryos (E1.1, E1.2, E1.7, E2.1—E2.3), however, this approach was not successful, likely due to inefficient removal of the genomic background during NGS library preparation and low amounts of viral RNA. Instead, we used the reptarenavirus genomes obtained from the parental animal to “fish out” i.e. to map the matching reads from the embryos, an approach we then also took for clutches 3 and 4. However, only scattered reads matching the parental viruses could be recovered from the NGS data for most embryos (S2 Table). Thus we decided to confirm the NGS findings by conventional RT-PCR using virus species-specific (VSS) primers, primers of our previous study [11] and primers designed based on the *de novo* assembled arenavirus genomes (primer sequences in S1 Table). For most clutches we also included additional samples, from tissues or cell cultures generated from the embryos, into the RT-PCR analysis (Tables 2 and 3).

**Table 3:** Detailed results obtained from the different specimens used for the RT-PCR approach to identify S segments (abbreviations and accession codes are given in S1 Table, and in materials and methods) in each animal in clutches 1–5. The virus genomes *de novo* assembled from NGS data of the parental sample and confirmed by VSS RT-PCR are indicated by virus abbreviations in bold, and viruses detected by VSS RT-PCR are shown in italics in parents and with + sign in the offspring. +/- sign indicates an ambiguous RT-PCR result.

Animals	Specimen	Viruses detected by virus species-specific (VSS) RT-PCR				
		S5-like	UGV-1 & -4	ABV-2	UGV-2 & -3	TSMV-2
**Clutch 1, Breeder 1**						
Mother	Brain	**S5-like**	**UGV-1 & -4**			
E1.1	Head	+	+			
E1.2	Body	+	**-**			
E1.6	CC (brain)	**-**	**-**			
E1.7	CC (brain)	**-**	+			
**Clutch 2, Breeder 2**						
Mother	Brain		**UGV-1 & -4**	**ABV-2**		
Father	Blood		**-**	**-**		
E2.1	Salpinx		+	+		
	CC (umbilicus)		**-**	+		
	CC (kidney)		+	**-**		
	CC (heart)		**-**	+		
E2.2	Body		**-**	**-**		
	CC (umbilicus)		+	+		
	CC (placenta)		**-**	**-**		
	CC (kidney)		+	+		
	CC (liver)		+	+		
E2.3	Body		**-**	**-**		
**Clutch 3, Breeder 1**						
Mother	Blood	**S5-like**			**UGV-2 & -3**	
PNA3.1	Brain	+			+	
	Kidney	+			+	
	Liver	+			**-**	
PNA3.2	Brain	+			+	
	Kidney	+			**-**	
	Liver	+			**-**	
PNA3.3	Brain	+			+	
	Kidney	+			**-**	
	Liver	+			**-**	
J3.1 (2 mo)	Liver	+			+	
	Kidney	+			+	
J3.2 (2 mo)	Liver	+			**-**	
	Kidney	+			**-**	
**Clutch 4, Breeder 1**						
Father	Blood				*UGV-2 & -3*	**TSMV-2**
	Brain				NA	NA
	Lung				**-**	+
Mother	Serum				**-**	**-**
PNA4.1	Placenta				**-**	+
	Kidney				**-**	+
	Lung				**-**	+
PNA4.2	Placenta				+	+
	Kidney				**-**	+
	Lung				**-**	+
	Brain				**-**	+
**Clutch 5, Breeder 1**						
Father	Liver	*S5-like*			*UGV-2 & -3*	
J5.8 (12 mo)	Brain	+			+	
J5.8 (12 mo)	Liver	+			**-**	
J5.9 (12 mo)	Liver	+			+	
J5.10 (12 mo)	Liver	+			+	
J5.11 (12 mo)	Liver	+			**-**	
J5.12 (12 mo)	Brain	+			+	
J5.13 (12 mo)	Brain	+			+	
J5.14 (12 mo)	Brain	+			**-**	
J5.15 (12 mo)	Brain	+			+	
J5.18 (12 mo)	Liver	+			+	

For the three embryos of clutch 1, the mapping yielded reads matching five (E1.1), two (E1.2) and three (E1.7) of the six L segments and both S segments (all embryos) identified in the mother. For the primary cell culture of E1.7, the reads each covered the entire segments, which might be a consequence of the higher virus content in the supernatant compared to the tissues which were examined for E1.1 and E1.2. The VSS RT-PCRs confirmed the presence of several to all parental L and S segments in the embryonal tissues (E1.1 and E1.2) and cultured brain cells (E1.6 and E1.7) (Tables 2 and 3).

For clutch 2, reads matching two L and both S segments were identified by mapping the NGS data of E2.1 (kidney cell culture), E2.2 and E2.3 (both tissue homogenates) against parental viruses (S1 Table). The VSS RT-PCRs confirmed the NGS findings and identified the parental L and S segments also in homogenates of salpinx and placenta and in cultured cells from umbilicus, placenta and organs (Tables 2 and 3).

For clutch 3, we identified reads matching three L and two S segments of the maternal viruses for two perinatal abortions (PNA3.1 and 3.3) and reads matching each two L and S segments for the third (PNA3.2) by the mapping approach. VSS RT-PCRs on samples from several organs (brain, kidney, liver) confirmed the NGS findings. They also identified maternal L and S segments in the liver and kidney of the juvenile snakes euthanized at the age of 2 months (Tables 2 and 3).

For clutch 4, the mapping approach yielded a few reads matching both the L and S segment of the virus identified in the father in one perinatal abortion (PNA4.1), and for the second (PNA4.2), only a single read matching the L segment. Since the subsequent VSS RT-PCR of the PNA samples yielded only a weak reaction for the TSMV-2 L segment, we then applied all L segment primers available from the different viruses to RNA extracted from paternal blood and lung, and from the maternal blood sample. Curiously, while the brain of the father remained positive for only a single virus, the blood contained a further 7 reptarenavirus L segments, three of which were also found in the maternal blood. VSS RT-PCRs then identified several paternal L and S segments in the tissues of both perinatal abortions (Tables 2 and 3).

**Fig 3:**
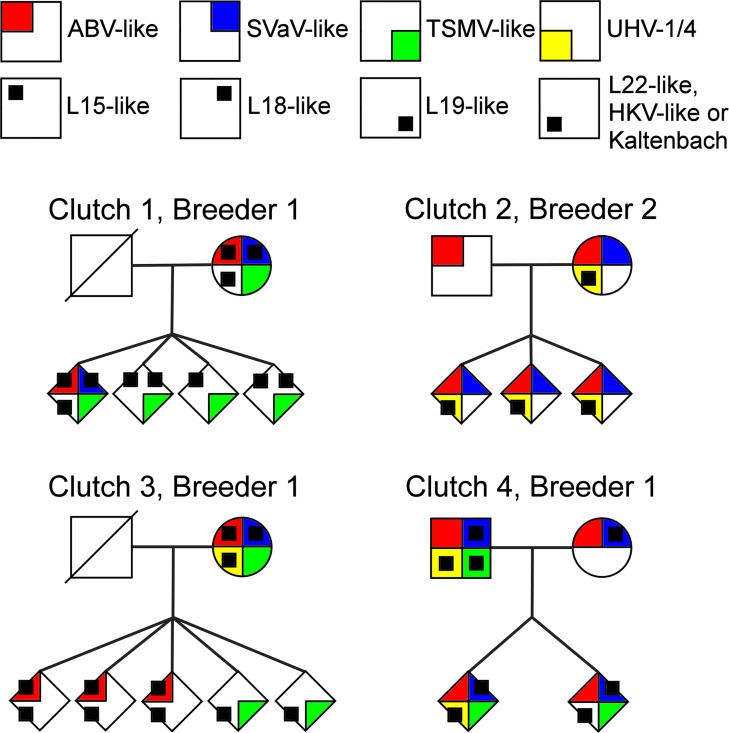
Vertical transmission of viruses presented in the form of a pedigree for the clutches with embryos and perinatal abortions. The viruses sequenced by NGS are indicated by different colours, no samples were available for the fathers of clutches 1 and 3 (indicated by crossed empty box).

Since the results obtained from clutches 1, 3 and 4 suggested that we had characterized the “reptarenavirome” of breeder 1’s collection, we did not perform NGS for clutch 5, but tested the father and several of his 12-month-old juvenile offspring, which were in the majority confirmed to suffer from BIBD based on the presence of viral IB and viral antigen in tissues, with all L and S segment VSS RT-PCRs of the present and an earlier study [11]. The father was positive for four of these viruses, and the juveniles were all found to carry at least two of their father’s L segments (Tables 2 and 3). The results for the L segment VSS RT-PCRs for each clutch are summarized in Fig 3. The raw data for VSS RT-PCRs are shown in S1–S5 Figs (S1 Fig, S2 Fig, S3 Fig, S4 Fig and S5 Fig).

## Discussion

So far, studies on the transmission of reptarenaviruses are scarce, and transmission via direct contact, through droplets or aerosols, or via vectors has been discussed [1, 2]. In this study on naturally infected captive animals we combined classical and more modern techniques and could demonstrate that reptarenaviruses and BIBD can be vertically transmitted. The study included five *B*. *constrictor* clutches with BIBD-positive parental animals, and by NGS combined with *de novo* genome assembly we could retrieve nearly complete reptarenavirus L and S segments in three of the four studied parental snakes. Because the different L segments identified in the parental animals were <76% identical to each other, we interpreted their identification as evidence of reptarenavirus co-infection. We could further show that co-infecting reptarenaviruses are often co-transmitted vertically from parents to offspring. By combining NGS and virus species-specific (VSS) RT-PCRs we could confirm the vertical transmission(s) and show that the offspring retains co-infecting viruses over a long period of time, i.e. for at least 12 months after birth.

Currently the strongest evidence of reptarenaviruses being the causative agents of BIBD is the fact that the IBs pathognomonic to BIBD [2] consist mainly, if not solely, of reptarenavirus NP [4, 5, 9, 14]. Although this does not rule out the possibility of another, yet unidentified, microbe [for example an (endogenous) retrovirus] contributing to the development of the disease, it clearly demonstrates that reptarenavirus infection is a prerequisite for BIBD. In the embryos, reptarenavirus infection was not associated with IB formation; however, viral antigen was found in occasional cells in brain, liver and kidneys. Furthermore, primary cell cultures derived from embryos of BIBD positive mothers promoted (part of) the maternal reptarenavirome and also showed viral antigen expression. IB formation was seen in older offspring, first in one of the PNA, consistently in all virus genome-positive juveniles from 2 months of age, confirming that reptarenavirus infection *in vivo* does indeed provoke all the characteristics of BIBD.

Vertical transmission occurs in the reservoir hosts of many arenaviruses. For example, LCMV and MACV can be transmitted transovarially [17, 18] and/or transplacentally [19]. Additionally, infection through semen or maternal blood has been suggested for MACV and Latino virus [20]. Prenatal infection plays an important role in virus maintenance, since for some arenaviruses (LCMV, MACV, and LASV) it may lead to chronic infections [21].

For reptarenaviruses, the precise mode of vertical transmission is not yet known, but our study provides evidence that the viruses of both mother and father can be passed to the offspring, and that the transmission can occur already early in gestation. We were able to isolate viruses also from cell cultures originating from placenta, salpinx, and umbilicus. Since the *B*. *constrictor* embryo does not get into contact with the maternal blood, this indicates that transmission from the mother could also result from contact between maternal tissues and the chorioallantois. However, more detailed studies on the reproductive tract of snakes with BIBD are needed to elucidate the exact mechanisms of transmission from both the maternal and paternal animal.

The convention among snake breeders that also both breeders in our study followed is that the neonates are removed from the mother’s cage within a few hours. The clutch is then housed separately until the first shedding at 6–12 days of age, after which the animals are separated and housed in individual cages [30]. This, together with the strict hygiene rules that are applied, does not exclude transmission of viruses between siblings during their first days of life, but renders horizontal infection unlikely thereafter.

It was overall surprising to see how many offspring exhibited reptarenavirus infection without evidence of IB formation or viral antigen expression (4/5 perinatal abortions, one 2-month-old juvenile) or without IB formation and only occasional cells expressing viral antigen (all tested embryos, two 12-month-old juveniles), i.e. BIBD. Also, the fact that we found BIBD-negative animals to carry reptarenaviral RNA in the blood suggests that viremia may occur frequently, not only in association with the disease, but also in seemingly healthy animals. However, light microscopy and IH are comparatively insensitive methods, and thus the above findings could also be due to low level viral replication. Alternatively, our findings could indicate that reptarenavirus infection has a long incubation period, and that both endogenous and exogenous factors can influence the development of BIBD. It has recently been suggested that transient reptarenavirus infections can occur [31]. Although we cannot disprove this assumption, the fact that the vast majority of juvenile offspring from snakes with BIBD in our study eventually developed BIBD suggests that at least prenatal reptarenavirus infections generally persist. We recently observed that snakes with BIBD rarely exhibit anti-reptarenavirus antibodies [32]. This could indicate that prenatal infection results in tolerance to reptarenaviruses, allowing persistent infection. Chang and co-workers recently reported that the vast majority of reptarenavirus infected and BIBD positive *B*. *constrictors* are clinically healthy [14], which would be in line with the above hypothesis. Further studies are required to show if the hypothesis is correct and what determines the subsequent IB formation.

Our observation on the vertical transmission of co-infecting viruses sheds light on the potential evolution of reptarenaviruses. The research field of “reptarenavirology” is fairly young, and the taxonomical classification scheme of these viruses is yet to be determined. After the most recent report from the arenavirus study group of the International Committee on Taxonomy of Viruses (ICTV) [8] our group and the group of Stenglein and co-workers reported a multitude of complete L and S segments identified by NGS in tissues of snakes with BIBD [10, 11]. Both groups also observed a seemingly unbalanced ratio of L and S segments in a single individual, similarly to what we report herein. These findings are the challenge for the classification of reptarenaviruses. For the present report we followed the ICTV [8] and considered the different reptarenavirus L segments to derive from different reptarenavirus species when their nucleotide sequence identity was below 76%. We took this approach, because currently not all of the information required to fulfil all criteria of a virus species are available. We also hypothesize that in the past, i.e. before multiple cross-species transfers (between and from the unknown reservoir hosts), each L and S segment pair formed a definite, classifiable species. For mammarenaviruses it has been reported that the persistent infection of cell cultures with one mammarenavirus excludes the replication of homologous and antigenetically related viruses, but enables the growth of non-related mammarenaviruses [33]. Similarly, we have only identified L segments of different reptarenavirus species (based on the criteria above) in snakes with BIBD. Assuming that there is (or was) a reservoir host for each reptarenavirus species, it can be hypothesised that, with more relaxed hygiene regimens, housing different snake species in the same facilities has enabled cross-species mixing of the viruses. Co-infection might then have enabled the mixing of L and S segments, and reassortment, and vertical transmission of these persistently infecting viruses may have contributed to the plethora of reptarenaviruses that we now detect in captive boid snakes. The apparent existence of more viral L than S segments might be related to the fact that the S segment harbours the viral glycoproteins. As these are essential for host cell entry, the S segment that guarantees the most efficient gene transfer might be enriched during co-infections. The selection pressure on the S segment may further be enforced by the functions of the NP in viral replication [34]. If the L and S segments could pair more or less freely with each other, the selection pressure on the L segment (harbouring the RNA dependent RNA polymerase and the viral matrix protein) would be less strong. It is also possible that more than a single pair of L and S segments are packed inside the virion, which would render the taxonomical classification of reptarenviruses even more complex. Currently there is no data on the factors enabling or disabling the pairing of different L and S segments. Since protein-protein interactions, among other factors, contribute to the formation of progeny viruses and cell-cell transmission, we speculate that L and S segment pairing would not occur in a completely random fashion (otherwise one would expect to have roughly equal numbers of known L and S segments). Further studies are needed to tackle these questions and to prove or disprove the above hypothesis, which only represents a simplified version of reality.

In order to avoid infection and/or spreading of the disease within a collection, it would be essential to know all the factors behind reptarenavirus transmission. A six-month quarantine is generally recommended before a new animal is released into a collection, but whether this is sufficient to avoid reptarenavirus transmission is so far unknown [35]. The results that we obtained from clutch 5 indicate that it can take several months before a prenatally infected snake exhibits definite signs of BIBD. In any case, our results demonstrate that animals with BIBD/reptarenavirus infection should not breed, since the likelihood of offspring to become infected is high.

## Materials And Methods

### Ethics statement

All animals included into the study were snakes that were submitted by their owners to the Department of Veterinary Pathology, Vetsuisse Faculty, University of Zurich, Switzerland. They were euthanized according to ASPA, Animals (Scientific Procedures) Act 1986, schedule 1 (appropriate methods of humane killing, http://www.legislation.gov.uk/ukpga/1986/14/schedule/1) procedure and a full diagnostic post mortem examination was performed. Tissue samples from the dead animals were subjected to the different tests with owners' consent. The owners consented both to the euthanasia and the use of collected samples in this study. Because of suspected BIBD no ethical permissions were required for euthanasia nor the diagnostic-motivated necropsies (both routine veterinary purposes).

### Animals and sampling

The study was performed on five *B*. *constrictor* clutches from two breeders residing in Switzerland. The two breeders confirm that they have never exchanged animals with each other. All animals were examined for diagnostic purposes, i.e. BIBD diagnosis, upon the owners’ request, which was undertaken on a blood smear and/or by a full post mortem examination. Parental animals that were not euthanized were bled from the tail vein or by cardiac puncture to prepare a blood smear. For necropsy, animals were narcotized with CO_2_ followed by decapitation and immediate destruction of the brain by longitudinal sectioning. Immediately after euthanasia, a full post mortem examination was performed.

The following *B*. *constrictor* snakes were examined (Table 1); clutch 1: a BIBD-positive (blood smear) pregnant female (euthanized due to emaciation and poor general health) with seven embryos in the first third of gestation; clutch 2: a BIBD-positive pregnant female (euthanized due to the owner’s suspicion of illness and BIBD) with three embryos in the first third of gestation, the father was subsequently tested on blood smears; clutch 3: three perinatal abortions and two siblings euthanized at the age of two months for diagnostic purposes, blood tested from the mother for BIBD diagnosis; clutch 4: two perinatal abortions, blood tested from mother and father for BIBD diagnosis; clutch 5: 22 juveniles (seven euthanized at the age of eight months, eleven at 12 months, two at 18 months, two at 20 months) for BIBD diagnosis due to positivity of the father, euthanasia and post mortem examination of the father due to emaciation and chronic pyogranulomatous bacterial rhinitis. The clutch had been separated from the mother within 8 h after birth and individual animals housed separately since after the first shedding at 6–12 days of age.

From all necropsied animals, tissue samples were collected from a range of organs (brain, heart, lung, liver, pancreas, kidney, spleen and–in selected cases–spinal cord), fixed in 10% buffered formalin, and routinely paraffin wax embedded for histological and immunohistological examinations. Selected embryos were fixed and paraffin wax embedded in toto, others were subjected to RNA extraction and/or establishment of cell cultures (Table 1). For adult and juvenile snakes blood smears were prepared and air-dried for cytological examination, and the remaining blood was centrifuged at 1,000 x g for 5 min to separate serum and blood cells. The samples for RNA extraction and/or virus isolation were collected and frozen freshly at -80°C without fixative or processed immediately.

### Cytological, histological, and immunohistological examination

Blood smears were stained with May-Grünwald-Giemsa and a cytological examination was performed to determine the presence of the pathognomonic cytoplasmic IB within blood cells, as previously described [4]. From paraffin blocks, consecutive sections (3–5 μm) were prepared, stained with hematoxylin-eosin (HE) for the identification of the cytoplasmic IB, and subjected to immunohistological staining, using a rabbit anti-UHV NP antibody [15] for the demonstration of reptarenavirus antigen, as described [4]. Consecutive sections incubated with a non-reactive rabbit polyclonal antiserum served as negative controls.

### Cell cultures

From selected embryos (Table 1), samples of brain, heart, liver, kidney, umbilical cord and/or placenta were aseptically collected and subjected to tissue culture (30°C, 5% CO_2_), as described [4]. After passaging of the established cell cultures, aliquots of the cultures (cell-rich supernatants) were frozen at -80°C (Table 1). The established cell cultures were analysed at each passage and remained persistently infected throughout the study. The cell culture supernatants from the established cell cultures were used to inoculate permanent boid kidney cell cultures (I/1Ki [4]), for virus identification by NGS, and to prepare cell pellets for formalin fixation and paraffin wax embedding, followed by immunohistology for the detection of reptarenavirus antigen, as previously described [4]. Cell pellets prepared from uninfected control cells served as the negative, and cells infected with the UHV isolate [4] (containing both ABV-1 and UHV-1 [11]) as the positive controls.

### Sample preparation and reverse transcription-polymerase chain reaction (RT-PCR)

RNA was extracted from tissue samples with TRIzol or Trizol LS reagent (Life Technologies) utilizing mechanical homogenization with MagNA Lyser (Roche) following the manufacturer’s protocol. From cell culture supernatants (Table 1), RNAs were isolated with the QIAamp Viral RNA Mini Kit (Qiagen) according to the manufacturer’s instructions.

RNA isolation from blood samples (Table 1) was performed according to a modified protocol for avian blood [36]. Briefly, 100 μl of centrifuged, cell-enriched blood was mixed with 900 μl of TRIzol (or 250 μl of blood and 750 μl of Trizol LS) and homogenized through pipetting. After addition of chloroform and separation of the RNA containing phase by centrifugation (15 min, 12,000 x g, 4°C) the RNA was purified with the QiaGEN RNeasy Mini Kit (Qiagen) following the manufacturer’s protocol for RNA clean up.

The cDNAs were transcribed with random primers (1/6-1/10 of the isolated RNA was used as template) using either RevertAid Transcriptase or RevertAid Premium Transcriptase (both from Thermo Fisher Scientific), following the manufacturer’s recommendations for RNA transcription with random hexamer primers.

Phusion Flash High-Fidelity PCR Master Mix (Thermo Fisher Scientific) with 2 μl of cDNA as the template was utilized in the “initial” PCR and 1 μl in the VSS PCR reactions. The “initial” PCR, applied prior to obtaining the NGS data, was run using primers (Fwd 5’-GAGCACGTCCTGTGTGTGT-3’ and Rev 5’-GTGGTTGTGTATGGGAGAGG -3’) targeting an approximately 170 bp long fragment of the L segment of GGV (1199–1367), UHV (1201–1369), and Boa AV NL3 (1191–1359) in PCR amplifications with Phusion Flash High-Fidelity PCR Master Mix (Thermo Fisher Scientific) using the following cycling conditions: 1. initial denaturation 15 s at 98°C, 2. denaturation 1 s at 98°C, 3. annealing 5 s at 60°C, 4. extension 5 s at 72°C, steps 2 to 4 were repeated 38 times until final extension of 1 min at 72°C. For Sanger sequencing (performed by the DNA sequencing core facility of Medicum, University of Helsinki, Finland, or by Microsynth, Switzerland), the products of the “initial” RT-PCR were purified using either QIAquick PCR purification Kit (Qiagen) or QIAquick Gel Extraction Kit (Qiagen), both according to the manufacturer’s instructions. After NGS and *de novo* assembly (see below), virus species-specific (VSS) primers were designed and used to confirm the presence of viruses identified by NGS in parents and offspring. The VSS RT-PCRs (both the S and L segment primers are listed in S1 Table) with Phusion Flash High-Fidelity PCR Master Mix (Thermo Fisher Scientific) and 1 μl of cDNA were run using the following cycling conditions: 1. initial denaturation 15 s at 98°C, 2. denaturation 1 s at 98°C, 3. annealing 5 s at 60°C, 4. extension 7 s at 72°C, steps 2 to 4 were repeated 38 times until final extension of 1 min at 72°C. The products were analyzed by agarose gel electrophoresis (gel strength 1.5–1.75%) with GelRed nucleic acid stain (Biotium) pre-cast to gels, the visualization of bands was done under UV-light. Samples from various tissues of different animals served as the negative controls for each primer pair. The raw data for VSS RT-PCRs are shown in S1–S5 Figs (S1 Fig, S2 Fig, S3 Fig, S4 Fig and S5 Fig).

### Next-generation sequencing (NGS), de novo assembly, and phylogenetics

The purified RNAs were treated with DNAse I (Fermentas), and re-purified using the GeneJET RNA purification kit (Thermo Fisher Scientific). The RNA was further cleaned using the Ribo-Zero Gold rRNA Removal Kit for Epidemiology (Illumina) according to the manufacturer’s protocol. The indexed (New England Biolabs) NGS libraries were prepared using the NEBNext Ultra RNA Library Preparation Kit (New England Biolabs) following the manufacturer’s instructions. The library quantification was done using the NEBNext Library Quant Kit for Illumina (New England Biolabs), and 291-bp paired-end reads of the pooled libraries were sequenced on an Illumina MiSeq (Illumina) using the MiSeq Reagent Kit v3 (Illumina). Removal of reads matching the host genome and *de novo* sequence assembly were performed using MIRA version 4.0.2. (http://mira-assembler.sourceforge.net/) on CSC (IT Center for Science Ltd., Finland) Taito supercluster. Chipster v.3.1.0. was applied for the generation of subsets and any other handling of the data [37]. The reptarenavirus genomes *de novo* assembled from the parents’ samples were used to map the reads matching reptarenaviruses from the offspring samples in Unipro UGENE 1.14.2. [38] utilizing the Bowtie2 [29] tool. The raw NGS data is publicly available through sequence read archive (SRA) under object IDs 5921116 to 5921133 with respective URLs http://www.ncbi.nlm.nih.gov/sra/5921116 to http://www.ncbi.nlm.nih.gov/sra/5921133.

Phylogenetic analyses were performed with the newly recovered sequences combined with representative reptarenavirus sequences obtained from the NIAID Virus Pathogen Database and Analysis Resource (ViPR) [39] through the web site at http://www.viprbrc.org/. Complete S segment nt sequences (S6-like clutch 1, KX527580; S5-like clutch 1, KX527581; ABV-2 clutch 2, KX527576; UGV-1 clutch 2, KX527577; S6-like clutch 3, KX527578; S5-like clutch 3, KX527579; TSMV-2 clutch 4, KX527575; ABV-1, KR870010; ABV-2, KR870018; Boa AV NL3, NC_023761; CASV, NC_018481; GGV, NC_018483; UHV-1, KR870011; UHV-1 (Hetzel et al.), NC_023766; UHV-2, KR870016; UHV-3, KR870019; UGV-1, KR870012; UGV-2, KR870015; UGV-3, KR870013; UGV-4, KR870014; S1, KP071530; S2, KP071541; S3, KP071630; S4, KP071474; S5, KP071599; S6, KP071673; S6A, KP071502; S6B, KP071501; S7, KP071578; S8, KP071509; S9, KP071671; S10, KP071558; S11, KP071559) were aligned with ClustalX [40]. The nt sequences (abbreviation, accession code: ABV-4 clutch 1, KX527594; TSMV-1 clutch 1, KX527595; HKV-1 clutch 1, KX527596; KePV-1 clutch 1, KX527597; BSV-1 clutch 1, KX527598; SVaV-2 clutch 1, KX527599; ABV-3 clutch 2, KX527583; KaBV-1 clutch 2, KX527584; SVaV-1 clutch 2, KX527585; UHV-3 clutch 2, KX527586; SVaV-2 clutch 3, KX527587; KMHV-1 clutch 3, KX527588; KePV-1 clutch 3, KX527589; UHV-4 clutch 3, KX527590; TSMV-2 clutch 3, KX527591; ABV-4-clutch 3, KX527592; GMV-1 clutch 3, KX527593; TSMV-2 clutch 4, KX527582; ABV-1, KR870021; ABV-2, KR870033; ABV-3, KR870025; Boa AV NL3, NC_023762; CAS virus, CASV, NC_018484; Golden Gate virus, GGV, KP071475; HKV-1, KR870028; SVaV-1, KR870024; TSMV-1, KR870026; UHV-1, KR870020; UHV-1 (Hetzel et al.), NC_023765; UHV-2, KR870030; UHV-3, KR870032; UHV-4, KR870027; UGV-1, KR870022; UGV-2, KR870029; UGV-3, KR870023; L1, KP071529; L2, KP071475; L3, KP071523; L4, KP071488; L5, KP071489; L6, KP071492; L7, KP071477; L8, KP071511; L9, KP071563; L10, KP071503; L11, KP071512; L12, KP071550; L13, KP071574; L14, KP071562; L15, KP071551; L16, KP071614; L17, KP071547; L18, KP071481; L19, KP071548; L20, KP071564; L21, KP071478; L22, KP071476) coding for the RNA-dependent RNA polymerase were aligned using amino acid translation guidance in Translator X [41] with the MAFFT algorithm. The GTR (general time reversible) model was used for nucleotide substitutions. Phylogenetic trees were reconstructed by the maximum-likelihood method in MEGA 6.06 with 1,000 bootstrap replicates.

## Supporting Information

S1 Table Virus names with corresponding abbreviations, GenBank accession numbers, and primer sequences used for L and S segment amplifications by RT-PCR. (XLSX)

S2 Table Coverage and the number of reads matching the *de novo* assembled L and S segments in each NGS sample. Mapping of the raw NGS data was done using Bowtie2.
(XLSX)

S1 Fig Raw data of virus species-specific (VSS) RT-PCRs for clutch 1. The RT-PCR products were separated by agarose gel electrophoresis with GelRed (Biotium) nucleic acid stain pre-cast to gels, the bands visualized under UV-light. The VSS RT-PCR products with L segment primers are presented in left-side panels and S segment primer products on right-side panels.
(TIF)

S2 Fig Raw data of virus species-specific (VSS) RT-PCRs for clutch 2. The RT-PCR products were separated by agarose gel electrophoresis with GelRed (Biotium) nucleic acid stain pre-cast to gels, the bands visualized under UV-light. The VSS RT-PCR products with L segment primers are presented in left-side panels and S segment primer products on right-side panels.
(TIF)

S3 Fig Raw data of virus species-specific (VSS) RT-PCRs for clutch 3. The RT-PCR products were separated by agarose gel electrophoresis with GelRed (Biotium) nucleic acid stain pre-cast to gels, the bands visualized under UV-light. The VSS RT-PCR products with L segment primers are presented in left-side panels and S segment primer products on right-side panels.
(TIF)

S4 Fig Raw data of virus species-specific (VSS) RT-PCRs for clutch 4. The RT-PCR products were separated by agarose gel electrophoresis with GelRed (Biotium) nucleic acid stain pre-cast to gels, the bands visualized under UV-light. The VSS RT-PCR products with L segment primers are presented in left-side panels and S segment primer products on right-side panels.
(TIF)

S5 Fig Raw data of virus species-specific (VSS) RT-PCRs for clutch 5. The RT-PCR products were separated by agarose gel electrophoresis with GelRed (Biotium) nucleic acid stain pre-cast to gels, the bands visualized under UV-light. The VSS RT-PCR products with L segment primers are presented in left-side panels and S segment primer products on right-side panels.
(TIF)
