# Supplementary figures and images for: Co-infecting Reptarenaviruses Can Be Vertically Transmitted in Boa Constrictor
Source: PLoS Pathog. 2017 Jan 23;13(1):e1006179. doi: 10.1371/journal.ppat.1006179 (PMC5289648; doi:10.1371/journal.ppat.1006179)

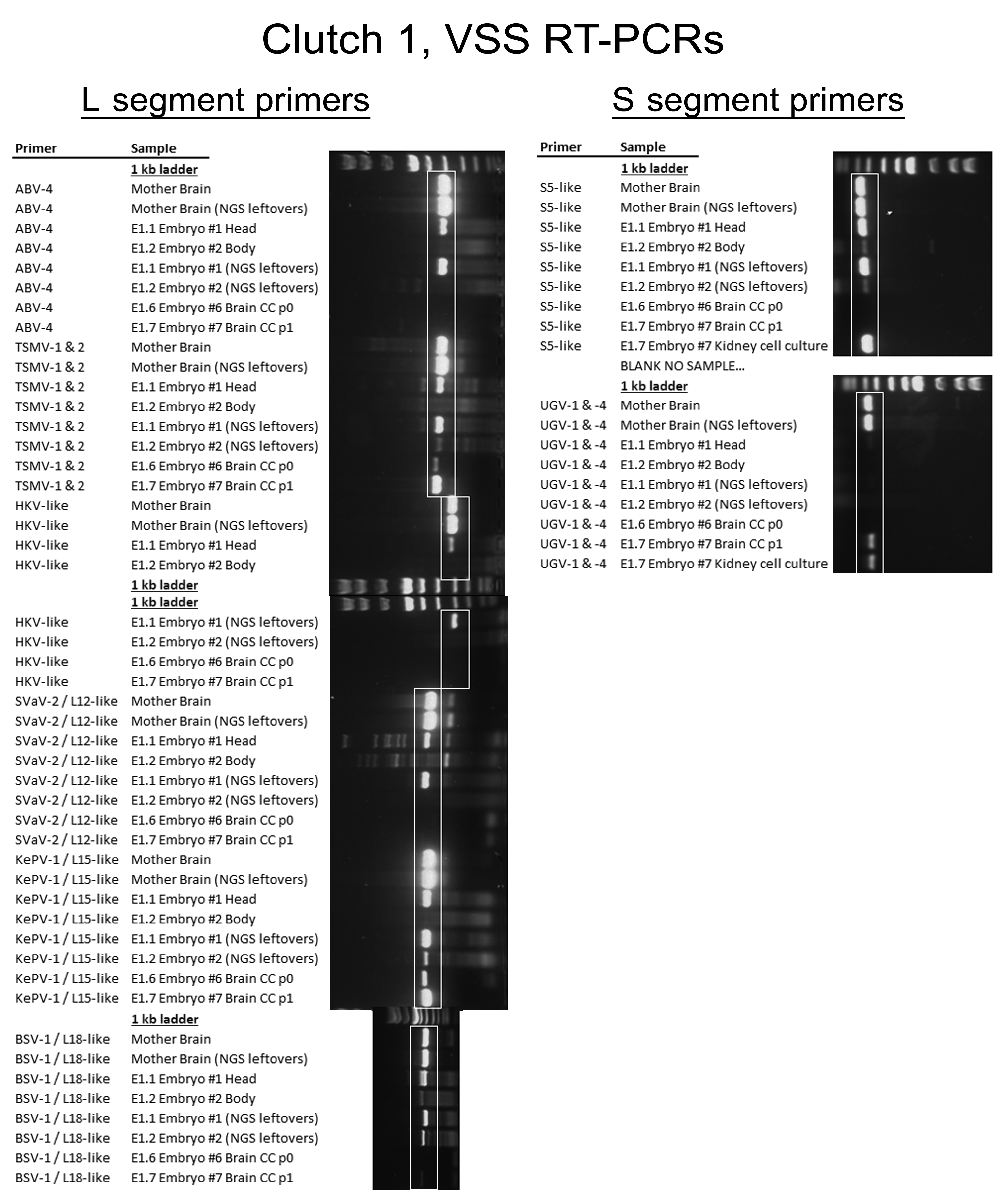

Supplement: S1 Fig — The RT-PCR products were separated by agarose gel electrophoresis with GelRed (Biotium) nucleic acid stain pre-cast to gels, the bands visualized under UV-light. The VSS RT-PCR products with L segment primers are presented in left-side panels and S segment primer products on right-side panels. (TIF) [file ppat.1006179.s003.tif]

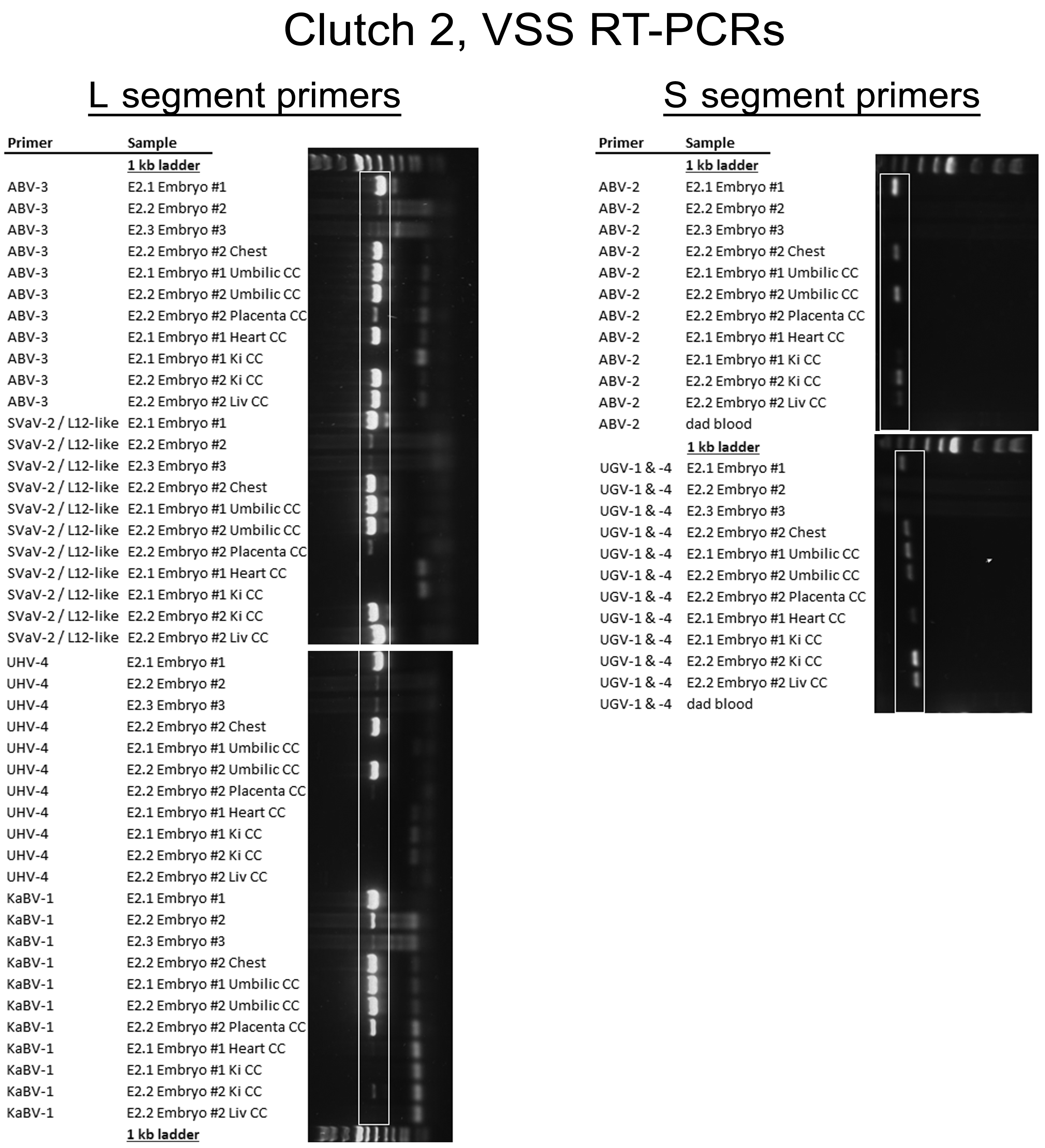

Supplement: S2 Fig — The RT-PCR products were separated by agarose gel electrophoresis with GelRed (Biotium) nucleic acid stain pre-cast to gels, the bands visualized under UV-light. The VSS RT-PCR products with L segment primers are presented in left-side panels and S segment primer products on right-side panels. (TIF) [file ppat.1006179.s004.tif]

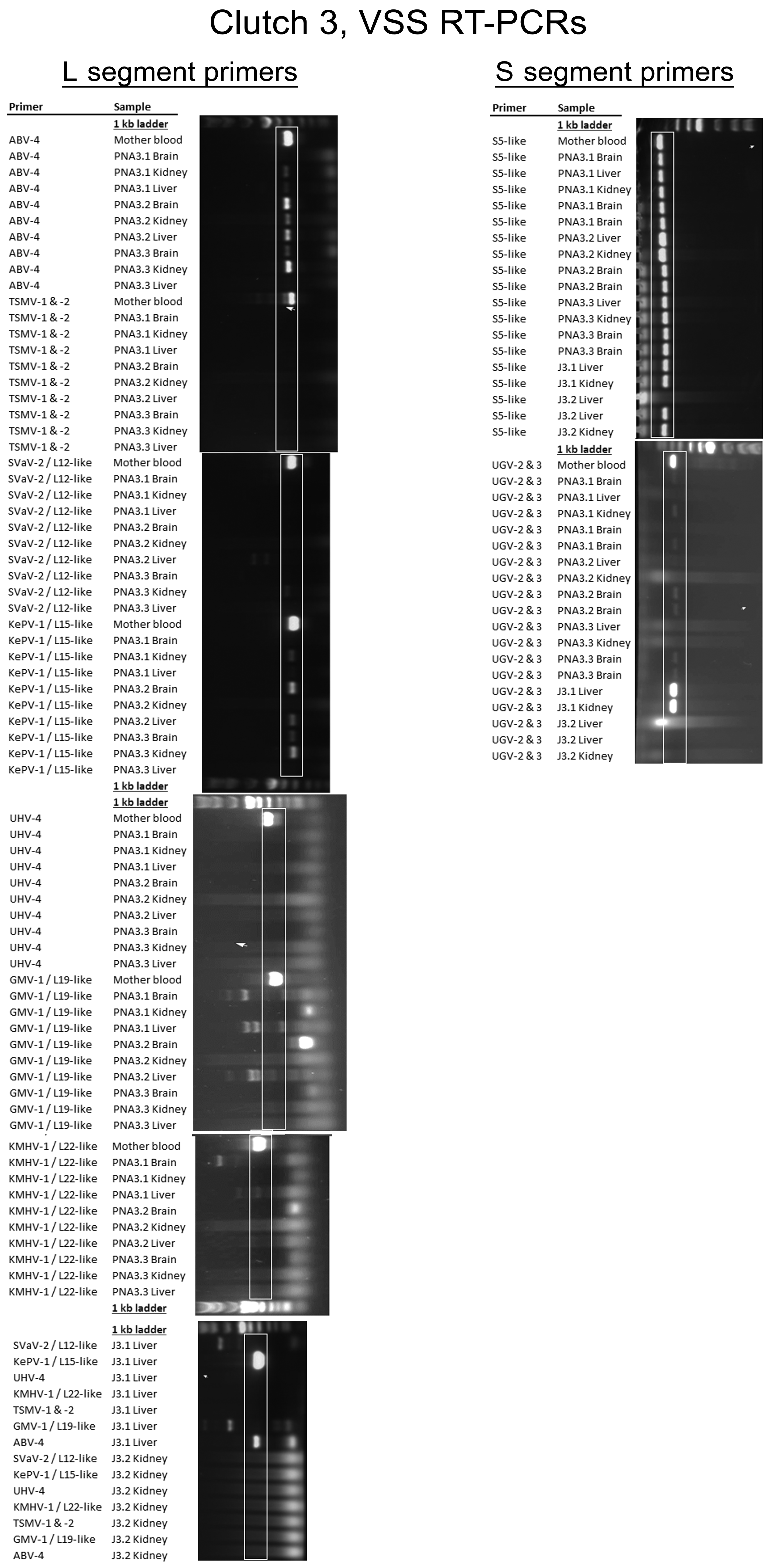

Supplement: S3 Fig — The RT-PCR products were separated by agarose gel electrophoresis with GelRed (Biotium) nucleic acid stain pre-cast to gels, the bands visualized under UV-light. The VSS RT-PCR products with L segment primers are presented in left-side panels and S segment primer products on right-side panels. (TIF) [file ppat.1006179.s005.tif]

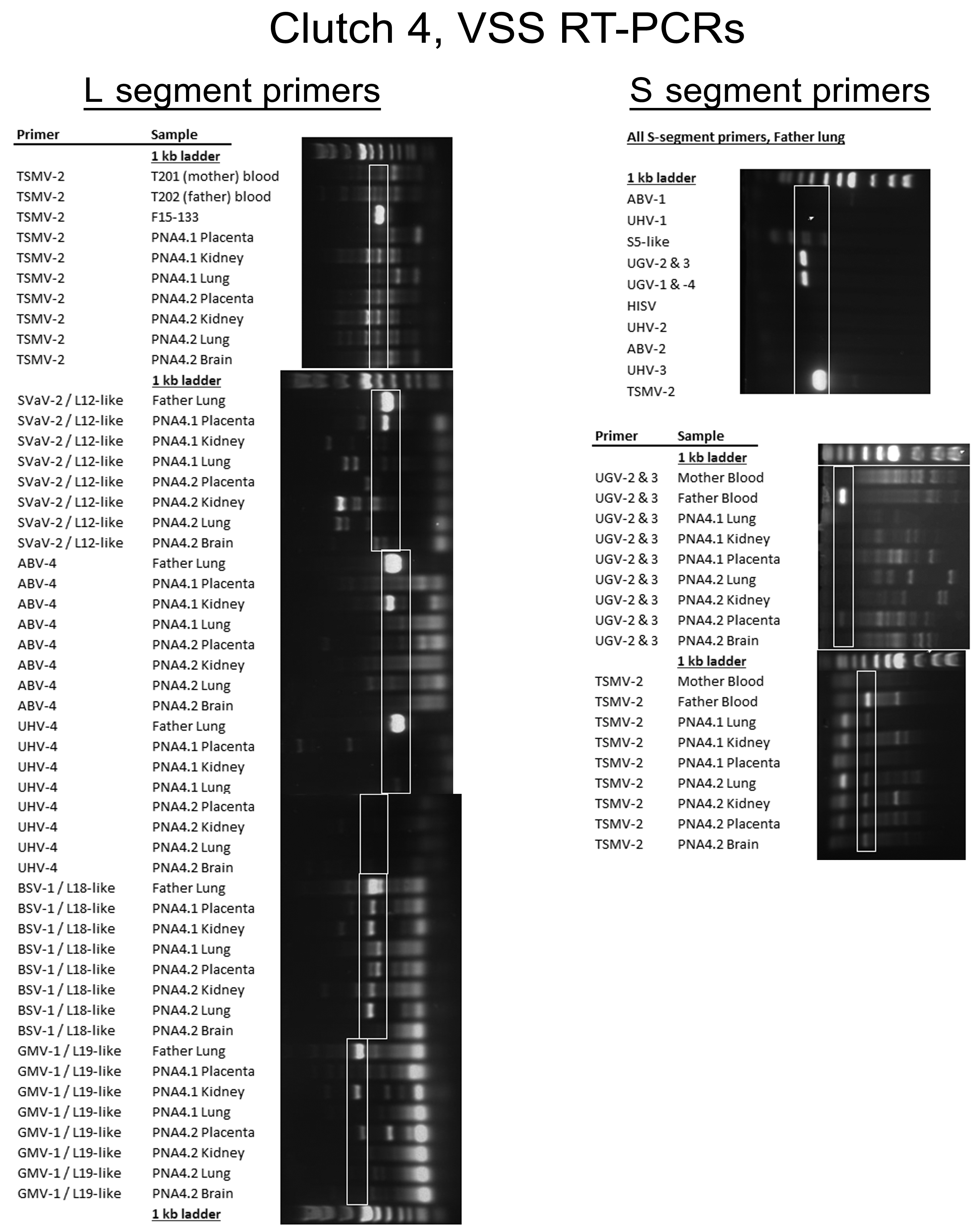

Supplement: S4 Fig — The RT-PCR products were separated by agarose gel electrophoresis with GelRed (Biotium) nucleic acid stain pre-cast to gels, the bands visualized under UV-light. The VSS RT-PCR products with L segment primers are presented in left-side panels and S segment primer products on right-side panels. (TIF) [file ppat.1006179.s006.tif]

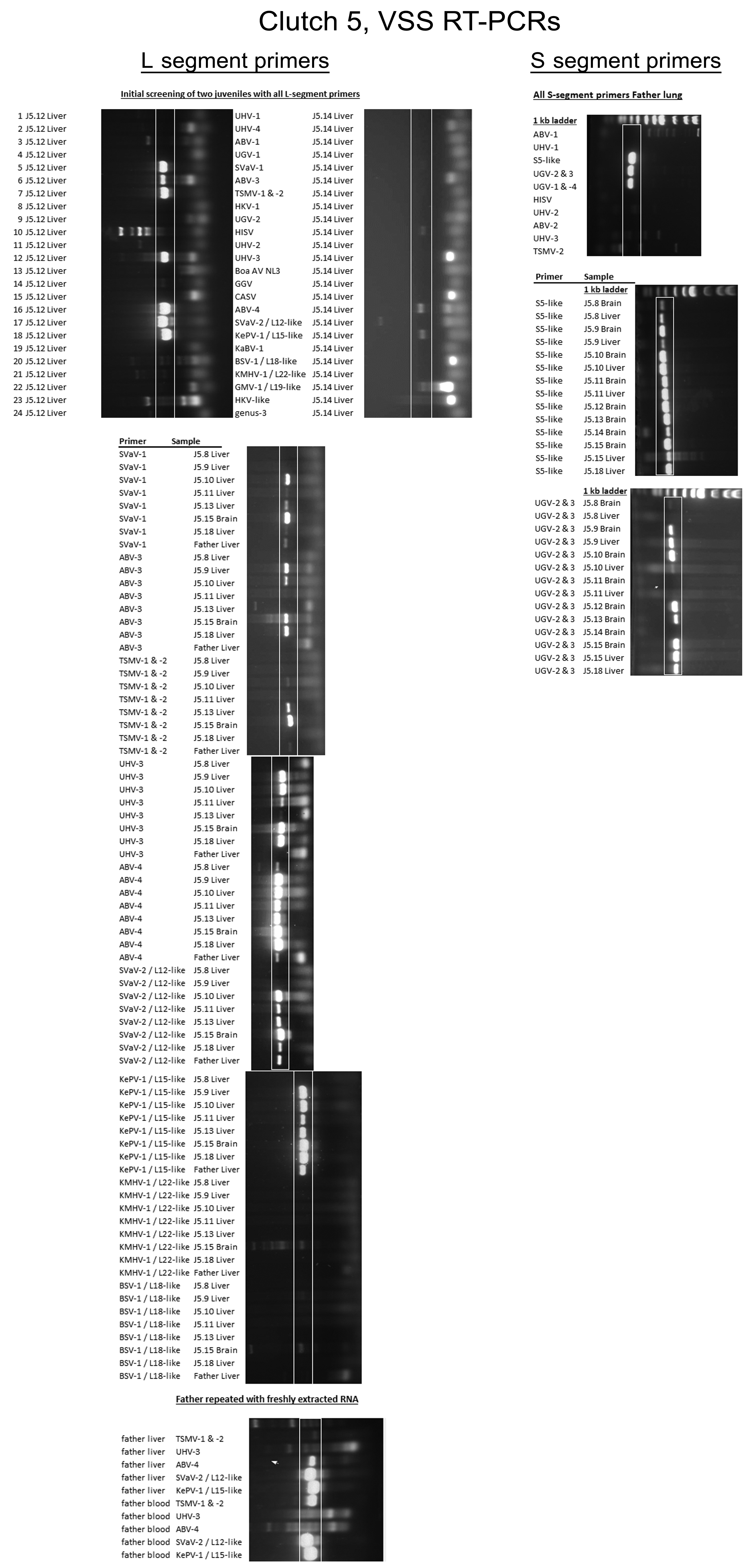

Supplement: S5 Fig — The RT-PCR products were separated by agarose gel electrophoresis with GelRed (Biotium) nucleic acid stain pre-cast to gels, the bands visualized under UV-light. The VSS RT-PCR products with L segment primers are presented in left-side panels and S segment primer products on right-side panels. (TIF) [file ppat.1006179.s007.tif]
